# Crystal structure and Hirshfeld analysis of di-μ-bromido-bis­[(1-{[dimeth­yl(phenyl­thio­meth­yl)sil­yl]meth­yl}piperidine-κ^2^*N*,*S*)copper(I)] and di-μ-iodido-bis­[(1-{[dimeth­yl(phenyl­thio­meth­yl)sil­yl]meth­yl}piperidine-κ^2^*N*,*S*)copper(I)]

**DOI:** 10.1107/S2056989026009862

**Published:** 2026-10-02

**Authors:** Kai Schäper, Michael Knorr, Carsten Strohmann

**Affiliations:** a TU Dortmund University, Institute for Inorganic Chemistry, Otto-Hahn-Strasse 6, 44227 Dortmund, Germany; b Université Marie et Louis Pasteur, Institute UTINAM UMR 6213 CNRS 16 Route de Gray 25030 Besançon France; Venezuelan Institute of Scientific Research, Venezuela

**Keywords:** crystal structure, dinuclear Cu^I^ complexes, iodine bridges, bromine bridges, organosilyl S,N donor

## Abstract

The use of 1-{[dimeth­yl(phenyl­thio­meth­yl)sil­yl]meth­yl}piperidine piperidine as chelating ligand has been probed by complexation on CuI and CuBr in MeCN solution to afford the dinuclear *S*,*N*-chelate title complexes, [Cu_2_I_2_(C_15_H_25_NSSi)_2_] and [Cu_2_Br_2_(C_15_H_25_NSSi)_2_], which feature a halide-bridged rhomboid-shaped Cu(μ_2_-*X*)Cu (*X* = I, Br) core with Cu⋯Cu contacts of 2.8249 (5) and 2.7631 (6) Å, respectively. The two isostructural complexes crystalize in the triclinic space group *P*1 with half mol­ecule in the asymmetric unit. An analysis of the Hirshfeld surfaces shows that H⋯H (van der Waals) inter­actions dominate with a contact percentage of over 80% in both complexes.

## Abstract 2

The dinuclear *S*,*N*-chelate title complexes feature a halide-bridged rhomboid-shaped Cu(μ_2_-*X*)Cu (*X* = I, Br) core with Cu⋯Cu contacts of 2.8249 (5) and 2.7631 (6) Å, respectively. The two isostructural complexes crystallize in the triclinic space group *P*1 with half mol­ecule in the asymmetric unit.

## 1. Chemical context

In previous work, we have investigated the potential of the [(piperidino­meth­yl)silylmeth­yl] fragment for organic transformations and the preparation of cyclo­metallated Pd^II^ complexes (Schildbach *et al.*, 2004 ▸), (Colquhoun, *et al.*, 2012 ▸), (Koller *et al.*, 2017 ▸). In this context we have also synthesized 1-{[dimeth­yl(phenyl­thio­meth­yl)sil­yl]methyl}piperidine, **1**, by treatment of the organosulfur compound (chloro­meth­yl)dimeth­yl[(phenyl­thio)­meth­yl]silane with piperidine (Koller *et al.*, 2017 ▸). Based on our experience on the coordination of dytopic ligands possessing both a hard *N*-donor site and a soft *S*-donor site according to the hard and soft acids and bases (HSAB) concept (Pearson, 1963 ▸), such as thio­ether functionalized 2-aza­butadienes (Jacquot-Rousseau *et al.*, 2005 ▸; Kinghat *et al.*, 2024 ▸; Kubicki *et al.*, 2025 ▸), we have chosen the soft copper(I) salts CuI and CuBr for the complexation studies. Cu^I^ is known to form mononuclear complexes with bidentate ligands (Kinghat *et al.*, 2016 ▸), but in most cases the dinuclear μ_2_-halide-bridged rhomboid dimer *L*_2_Cu(μ_2_-*X*)Cu*L*_2_ or higher nuclear clusters such as the Cu_4_*X*_4_ cubane cluster are formed (Kim *et al.*, 2007 ▸; Bovin *et al.*, 2023 ▸). For example, we reported recently on the structure of the rhomboid-shaped dimer [*L*_2_Cu(μ_2_-I)Cu*L*_2_] [*L*_2_ = (^*i*^PrS)_2_C=C(H)—N=CPh_2_] (CSD refcode IYEVAN; Kinghat *et al.*, 2024 ▸), in which the two copper centers are additionally coordinated by the sulfur and nitro­gen donor atoms in a chelating manner. This motif is also present in redox-active, tetra­dentate *S,N*-donor ligands that simultaneously coordinate both copper(I) centers (CSD refcode SAYJIO; Curcio *et al.*, 2017 ▸). The applications are not limited to redox chemistry; luminescence properties have also been observed for rhomboid Cu⋯Cu dimers upon excitation by visible light (Zhang *et al.*, 2009 ▸; Kobayashi *et al.*, 2013 ▸; Shou *et al.*, 2017 ▸). This luminescence is also observed for species embedded in supra­molecular assemblies, such as metallocages (Xu *et al.*, 2021 ▸).
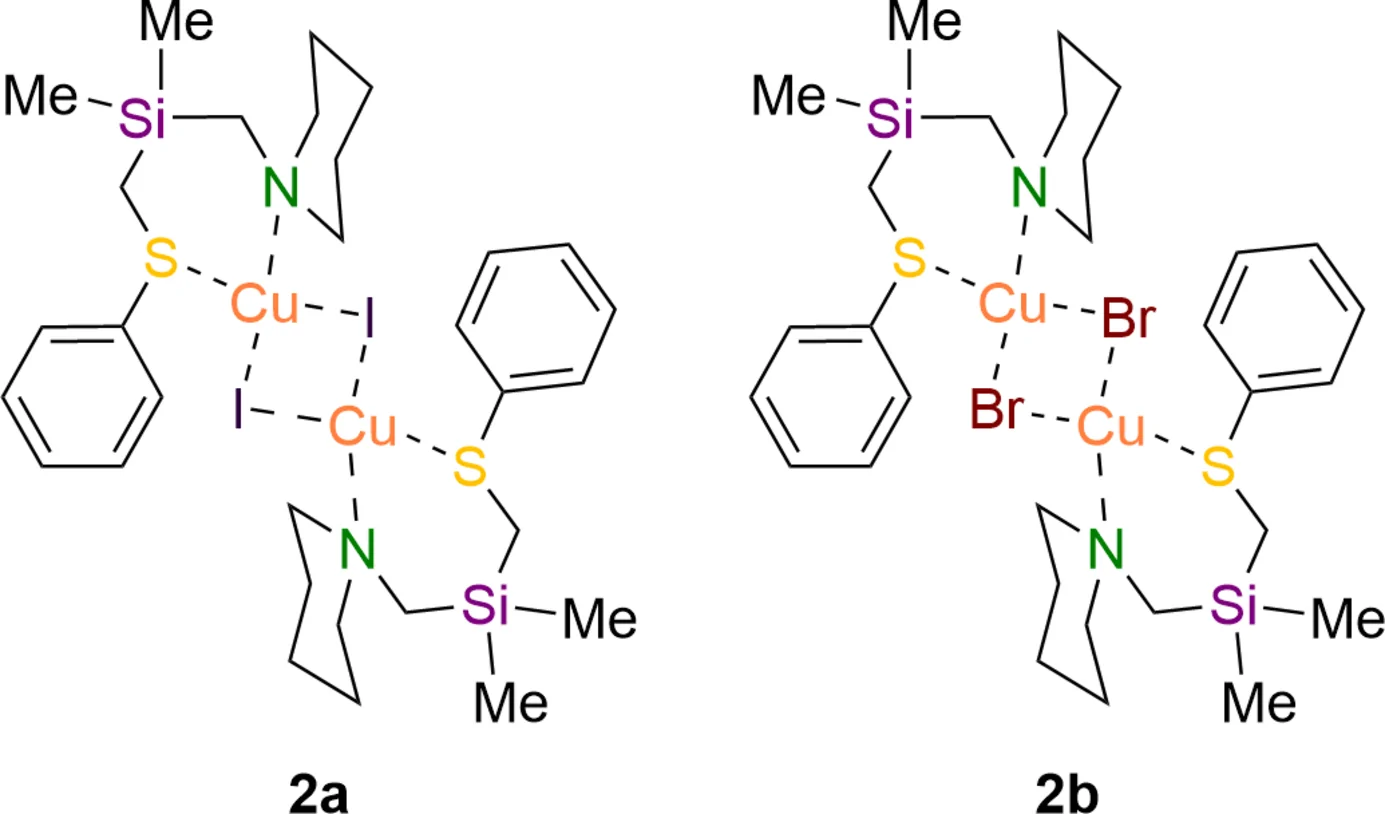


A characteristic feature associated with the formation of Cu⋯Cu polynuclear clusters is the observation of Cu⋯Cu distance below the sum of the van der Waals radii (2.8 Å; Bondi, 1964 ▸). This inter­action is referred to as ‘cuprophilicity’ and is indicative of an attractive inter­action between the copper(I) centers (Che *et al.*, 2000 ▸). The shortened inter­metallic distance can be enforced by bridging ligands such as μ_2_-halides, but can also occur in a ligand-unsupported fashion (Siemeling *et al.*, 1997 ▸; Cotton *et al.*, 1988 ▸). While most *S,N*-chelating ligands are based on thio­ether and pyridine or imine functionalities (Curcio *et al.*, 2017 ▸; Cheon *et al.*, 2013 ▸; Zhang *et al.*, 2009 ▸; Kinghat *et al.*, 2024 ▸), we report here two examples of rhomboid copper(I) halide dimers coordinated by organosilicon-bridged *S,N*-donor ligands, utilizing amine moieties as *N*-donors. In the herein reported examples of silicon-bridged ligands, the silicon center enhances the coordination ability of the donor sites by increasing the electron density of their neighboring carbon centers (Schneider *et al.*, 2023 ▸; Chen *et al.*, 2013 ▸; Khatyr *et al.* 2026 ▸).

## 2. Structural commentary

**Figure 1:**
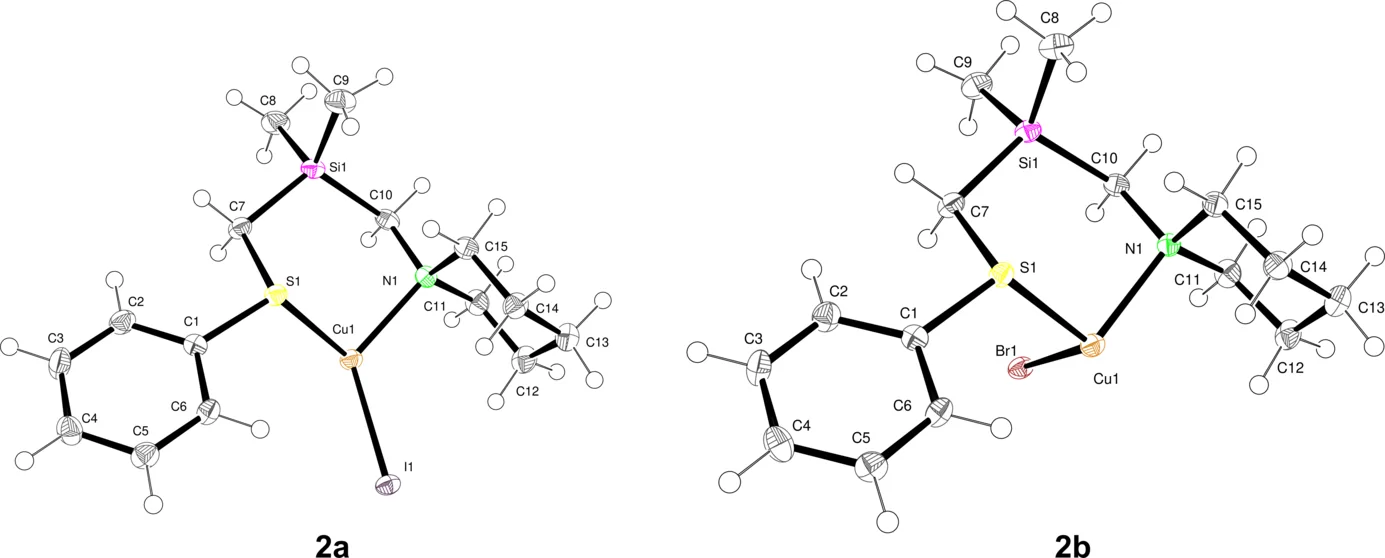
Asymmetric units of **2a** and **2b** with displacement ellipsoids drawn at the 50% probability level.

The title compounds Cu_2_I_2_[PhSCH_2_Si(Me_2_)CH_2_Pip]_2_ (**2a**) and Cu_2_Br_2_[PhSCH_2_Si(Me_2_)CH_2_Pip]_2_ (**2b**) crystallize at room temperature from aceto­nitrile in the centrosymmetric space group *P*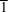 with half a mol­ecule in the asymmetric unit. The asymmetric units of the complexes are shown in Fig. 1 ▸, and characteristic bond parameters are listed in Tables 1 ▸ and 2 ▸. Both structures feature the same ligand, 1-{[dimeth­yl(phenyl­thio­meth­yl)sil­yl]meth­yl}piperidine, which comprises an amine and a thio­ether Lewis donor moiety bridged *via* an SiMe_2_ group as spacer. The S1—C7 bond length of 1.792 (2) Å and 1.798 (2) Å and the N1—C10 bond lengths of 1.483 (3) Å and 1.489 (3) Å for **2a** and **2b**, respectively, are consistent with typical values reported for such bond environments (Allen *et al.*, 1987 ▸). Slight deviations from the ideal tetra­hedral angle (109.5°) are observed around the nitro­gen atom. In compound **2a**, the bond angles around N1 range from 107.69 (16) to 113.40 (13)° while in compound **2b** they vary between 107.77 (16) and 112.46 (13)°. The N1—Cu1—S1 bite angles of 97.68 (5)° (**2a**) and 98.95 (5)° (**2b**) contribute to a slightly distorted tetra­hedral geometry around the Cu^I^ centers. The *t*_4_ parameters (Yang *et al.*, 2007 ▸) of 0.87 (**2a**) and 0.88 (**2b**) indicate a low degree of distortion in the tetra­hedral coordination environment, in contrast to other reported examples of this structural motif. The maximum *t*_4_ of 1.00 describes an ideal tetra­hedral coordination environment, whereas a *t*_4_ of 0 indicates an ideal square-planar coordination environment. A common coordination pattern in Cu^I^ halides is the formation of rhomboid dimers, which can exhibit a dissymmetric bridg­ing by the halides (Kinghat *et al.*, 2014 ▸). However, in both reported compounds a torsion angle of 0°, enforced by inversion symmetry at the center of the dimer, prevents this dissymmetry. A notable difference between the two structures lies in the Cu⋯Cu distances, which amounts to 2.8249 (5) Å for compound **2a**, while in compound **2b** it is slightly shorter at 2.7631 (6) Å. The latter value falls below the sum of the van der Waals radii of the two copper(I) centers (2.8 Å; Bondi, 1964 ▸), suggesting the presence of a weak Cu⋯Cu inter­action.

## 3. Supra­molecular features

**Figure 2:**
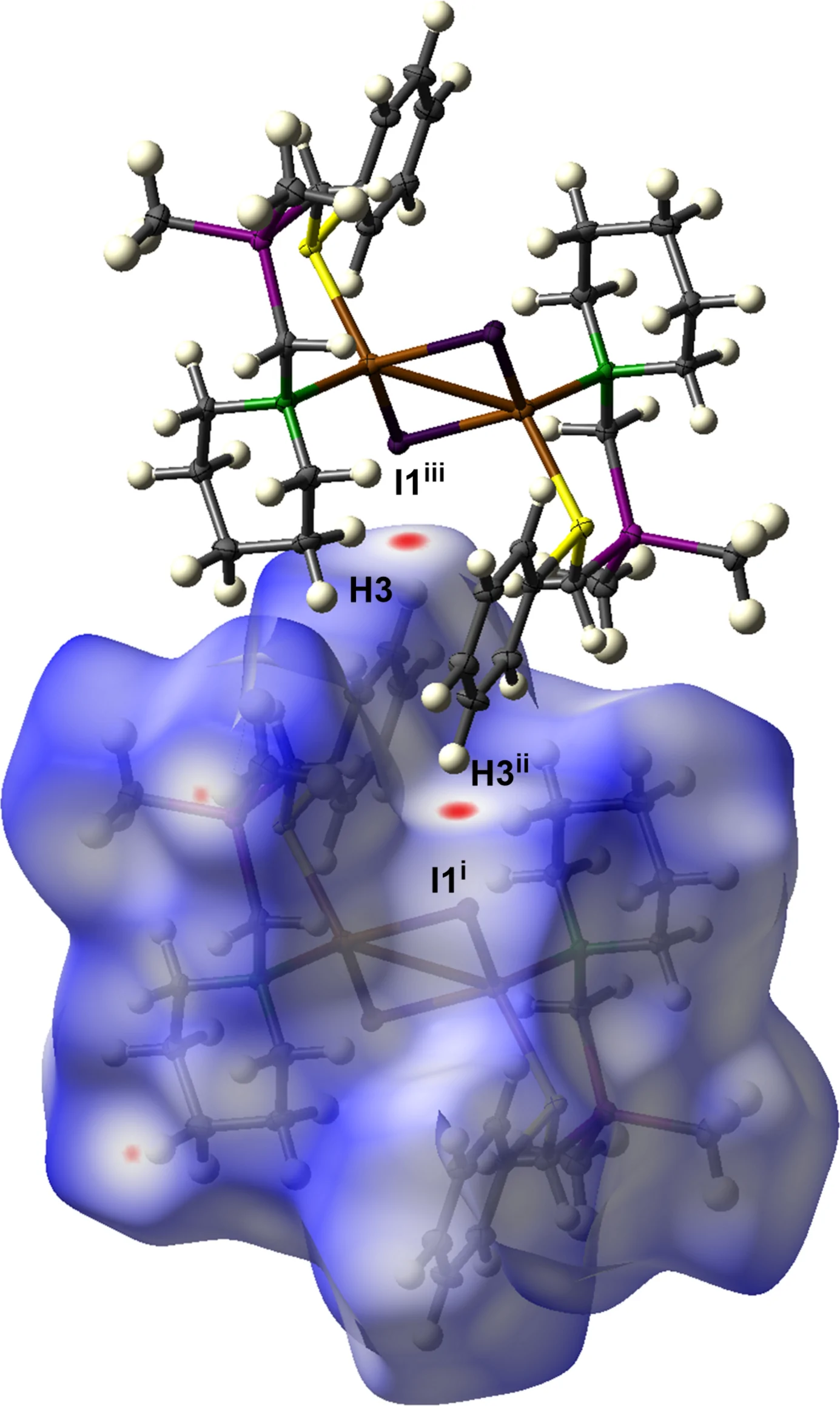
Hirshfeld surface analysis of **2a** showing close contacts in the crystal. The H3⋯I1^iii^ and I1^i^⋯H3^ii^ contacts are indicated by the red spots [symmetry codes: (i) −*x*, 1 − *y*, 1 − *z*; (ii) −*x*, 2 − *y*, 1 − *z*; (iii) *x*, 1 + *y*, *z*].

**Figure 3:**
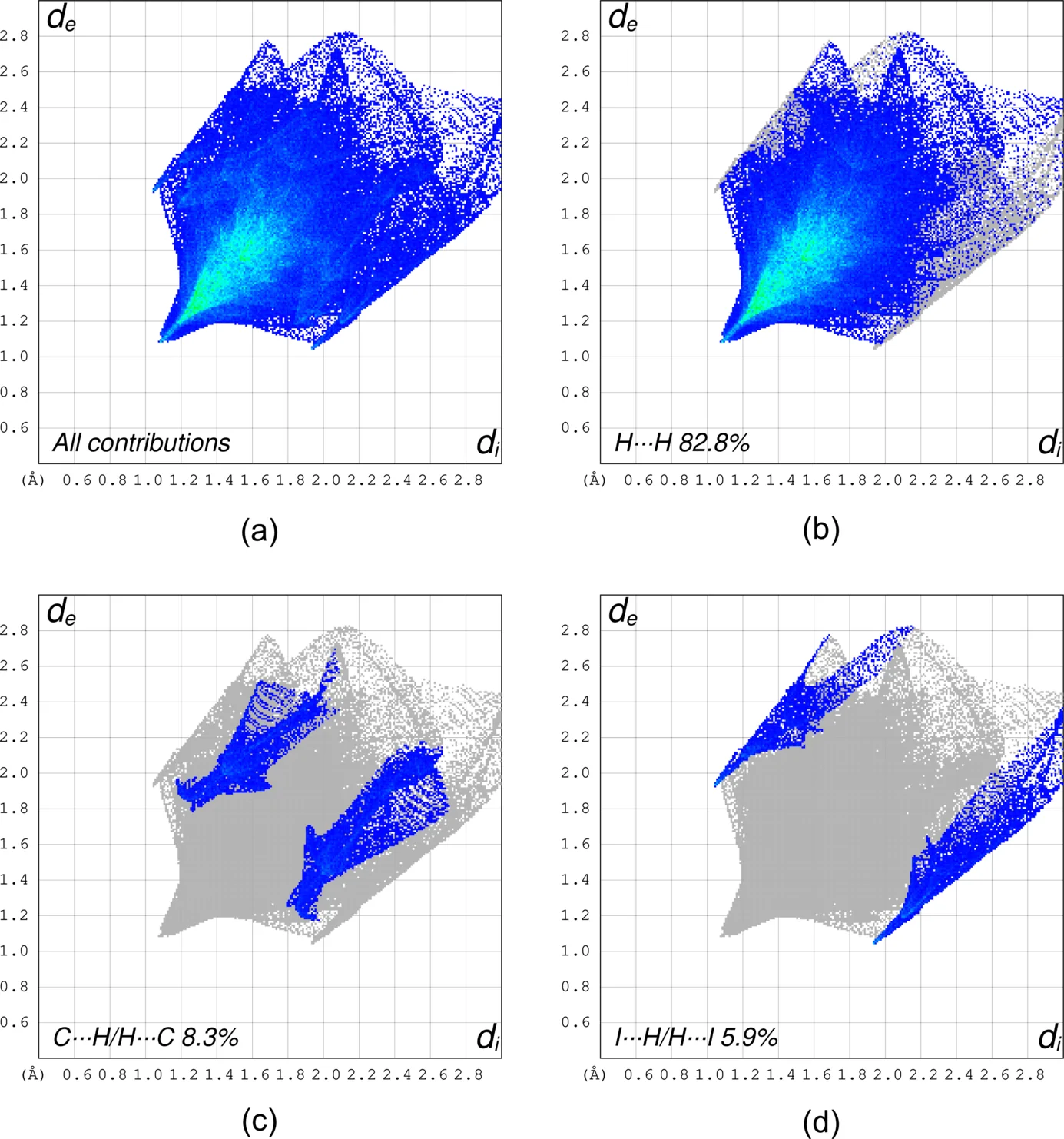
Two-dimensional fingerprint plots for compound **2a** showing (*a*) all contributions, and those delineated into (*b*) H⋯H, (*c*) C⋯H/H⋯C and (*d*) I⋯H/H⋯I-inter­actions.

**Figure 4:**
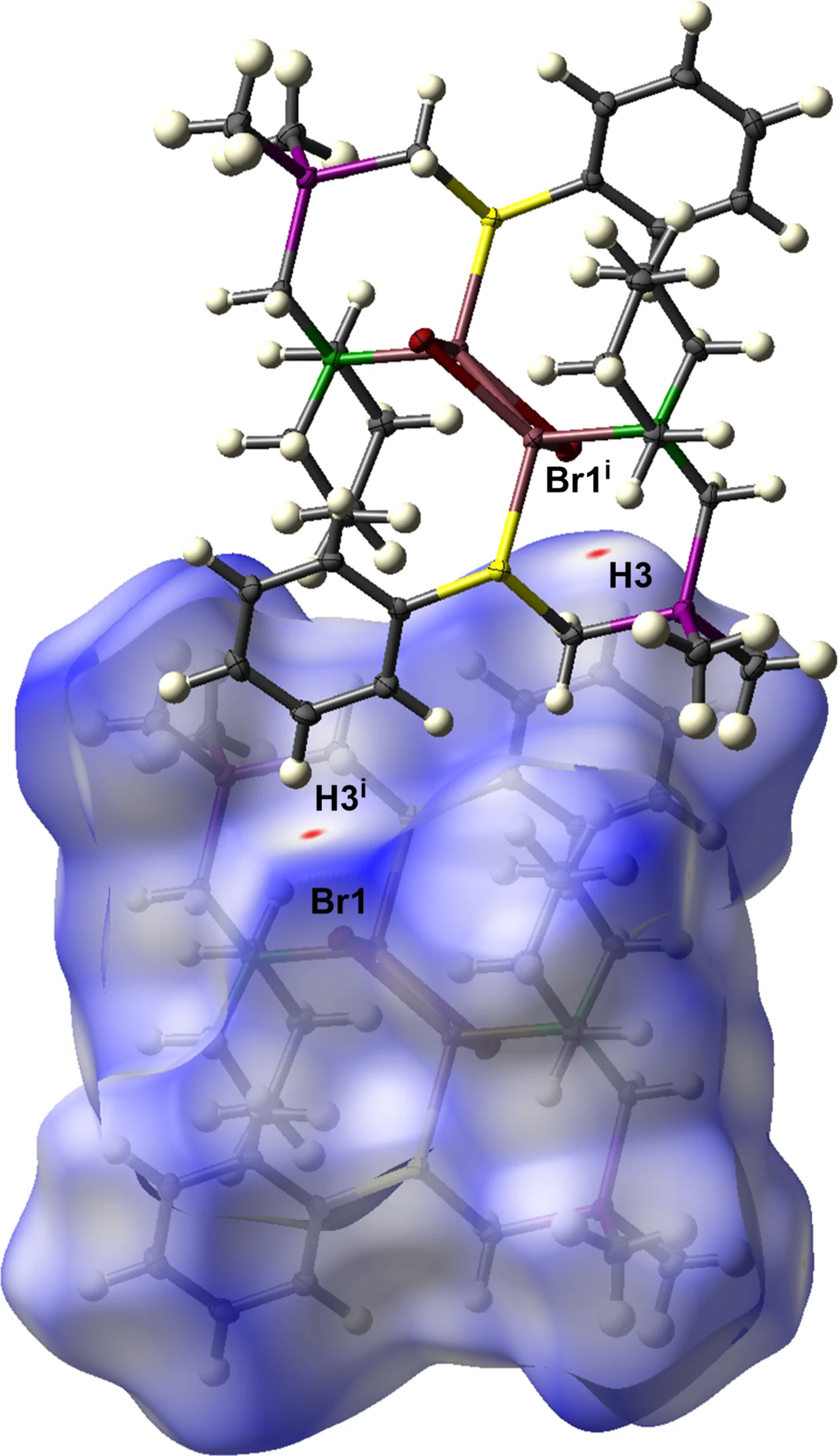
Hirshfeld surface analysis of **2b** showing close contacts in the crystal. The H3^i^⋯Br1 and Br1^i^⋯H3 contacts are indicated by the red spots [symmetry code: (i) 1 − *x*, 1 − *y*, 2 − *z*].

**Figure 5:**
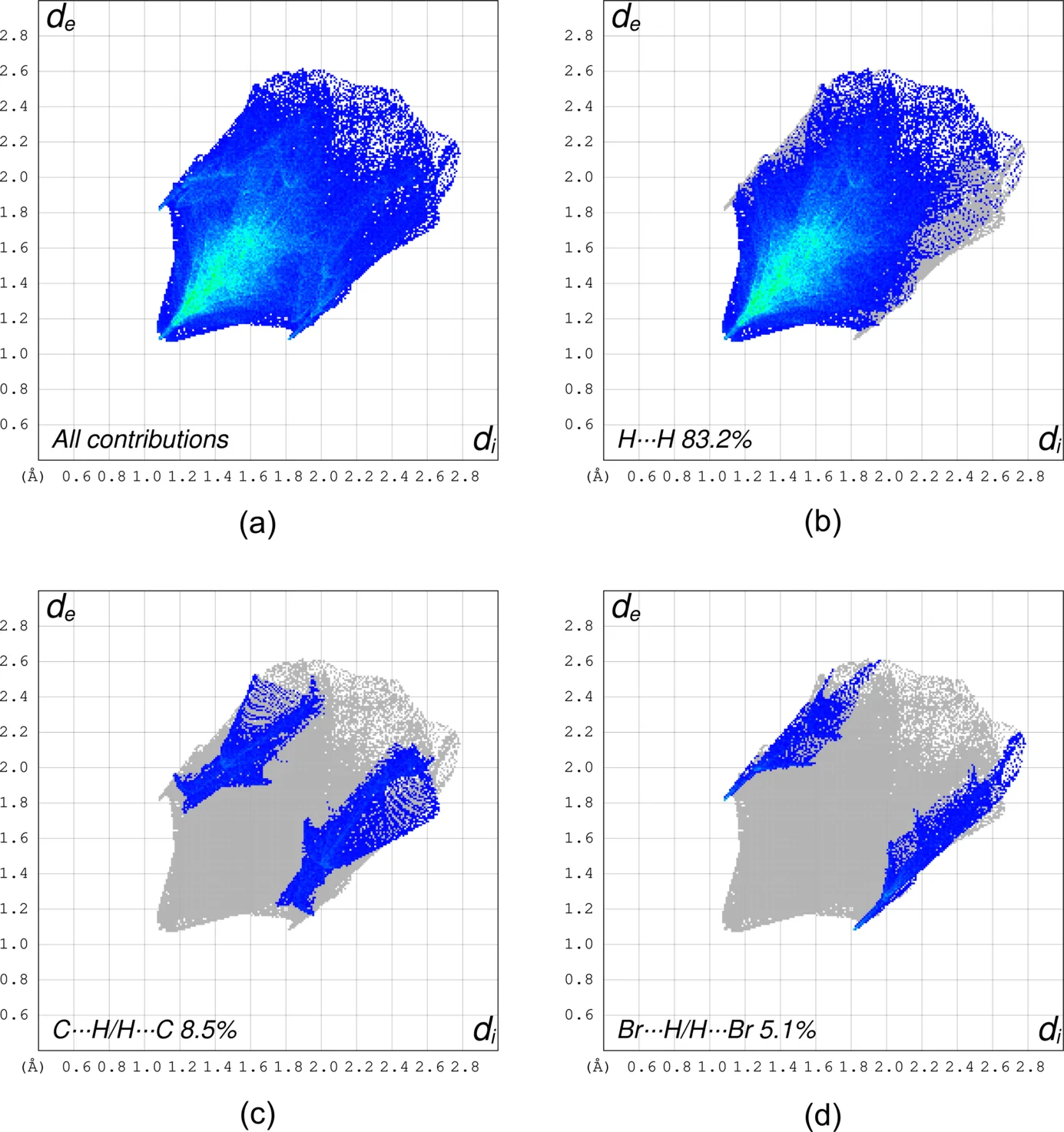
Two-dimensional fingerprint plots for compound **2b** showing (*a*) all contributions, and those delineated into (*b*) H⋯H, (*c*) C⋯H/H⋯C and (*d*) Br⋯H/H⋯Br-inter­actions.

**Figure 6:**
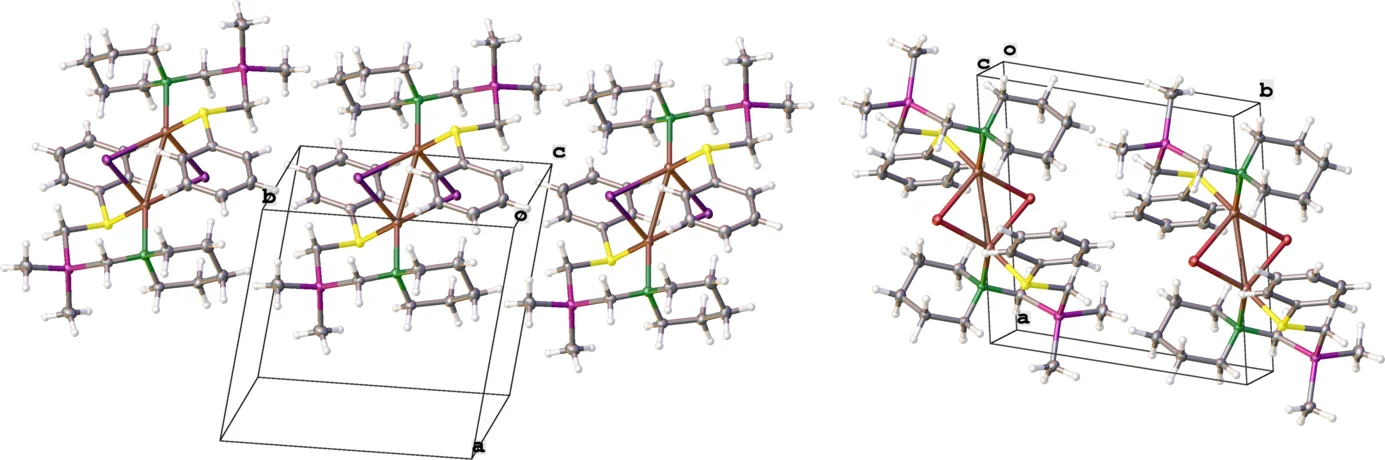
The packing of the solid state structures of **2a** (left) and **2b** (right) along the *b* axis.

For a detailed insight into the crystal packing, a Hirshfeld surface analysis was performed (Spackman & Jayatilaka, 2009 ▸). The Hirshfeld surface and the corresponding two-dimensional fingerprint plots were generated and analysed using *CrystalExplorer21* (Spackman *et al.* 2021 ▸). The Hirshfeld surface was mapped over *d*_norm_ in the range of −0.0729 to 1.4226 arbitrary units for compound **2a** and −0.0255 to 1.5298 arbitrary units for compound **2b**. While the Hirshfeld surfaces are shown in Fig. 2 ▸ ▸ (**2a**) and Fig. 4 ▸ (**2b**), the contribution of the different inter­molecular inter­actions are depicted in the two-dimensional fingerprint plots Fig. 3 ▸ (**2a**) and Fig. 5 ▸ (**2b**). Inter­molecular inter­actions are represented by the color coding on the surface: red spots indicate close contacts, whereas blue and white regions correspond to areas of no significant inter­action. Both title compounds exhibit a close contact between the halide center and the hydrogen center H3 of a neighboring mol­ecule H3⋯I1/I1⋯H3 within compound **2a** and H3⋯Br1/Br1⋯H3 within compound **2b** as can be seen from the Hirshfeld surfaces. Although these contacts are marked as close contacts on the surface, their contribution to the overall surface inter­actions is relatively small 5.1% for H⋯Br/Br⋯H and 5.9% for H⋯I/I⋯H making them comparable in magnitude. The largest contribution to the inter­actions in both compounds arises from weak van der Waals H⋯H contacts: 82.8% in compound **2a** and 83.2% in compound **2b**. This is followed by C⋯H/H⋯C inter­actions, contributing to 8.3% and 8.5%, respectively. The smallest contribution comes from S⋯H/H⋯S inter­actions at 3.1% in **2a** and 3.0% in **2b**. The crystal packing of both title compounds is illustrated in Fig. 6 ▸ and is characterized by propagation along the *b*-axis, which is promoted by the aforementioned close H⋯I/I⋯H inter­actions.

## 4. Database survey

A search of the Cambridge Crystallographic Database (Groom *et al.*, 2016 ▸; WebCSD September 2025) revealed several structures of rhomboid-shaped dimeric CuI complexes, in which the metal centers are inter­nally bridged by halide anions and additionally coordinated by *S,N*-donor ligands. The most common nitro­gen donors in these complexes are imine moieties, as observed in structures such as (CSD AXEWIL, AXEWOR; Flörke *et al.*, 2016 ▸) and (CSD BURYOE; Flörke, 2015 ▸). Note that for dimer AXEWOR (bis­{*N*-[2-(benzyl­sulfan­yl)phen­yl]-1,3-di­methyl­imidazolidin-2-imine}­bis­(μ-iodo)-dicopper) featuring a particular short Cu⋯Cu bond of only 2.5492 (4) Å, a deformation of the planar Cu(μ_2_-I)Cu core to a butterfly-shaped cluster is noticed, the μ_2_-I atoms forming the wing tips (Flörke *et al.*, 2016 ▸). Aromatic pyridine-type and nitrile-based nitro­gen donors have also been employed in similar coordination environments (CSD BARGARF; Lee *et al.*, 2017 ▸, CSD JIZZDE; Raghuvanshi *et al.*, 2019 ▸). Depending on the reaction conditions, CuI is reported to form with *t*-butyl­sulfan­yl)eth­yl)pyridine the dinuclear species bis­(μ_2_-iodo)­bis­{2-[2-(*t*-butyl­sulfan­yl)eth­yl]pyridine}­dicopper or a hepta­nuclear pinwheel-shaped Cu_7_I_7_ cluster (CSD XENZIB; Cheon *et al.*, 2013 ▸). The entries are dominated by discrete dinuclear [(*S,N*)Cu(μ_2_-I)Cu(*S*,*N*)] species, but also some macrocyclic scaffolds such as tetra­kis­(μ_2_-iodo)-bis­{μ_2_-4,6-bis­[(pyrid-2-ylmeth­yl)sulfanylmeth­yl]dibenzo(*b*,*d*)furan}­tetra­copper are known (CSD EHOFEM; Caradoc-Davies & Hanton, 2003 ▸). Using polydentate ligands such as 2,3-bis­(*t*-butyl­thio­meth­yl)quinoxaline also allows the assembly of supra­molecular networks incorporating the Cu(μ_2_-I)Cu motif (CSD NUHQOX; Zhang *et al.*, 2009 ▸). According to the database survey, only one example exists in which a secondary amine-based *S,N*-donor ligand is coordinated to an iodine-bridged copper(I) dimer (CSD TOTQAU; Cran *et al.*, 1996 ▸). The Cu⋯Cu distances reported for the two independent mol­ecules of bis­(μ_2_-iodo)-bis­[*N*-methyl-2-(*t*-butyl­thio)-1-phenyl-ethyl­amine-*N,S*]dicopper [2.587 (1) and 2.650 (2) Å] are considerably shorter than those of **2a.** Likewise, no reports were identified involving organosilicon-bridged mixed *S,N*-donor ligands coordinating the mentioned copper(I) dimers. A variation of this structural motif, replacing a *N*-donor moiety with a second thio­ether, leads to the formation of another organosilicon-bridged ligand capable of chelating the targeted copper(I) dimer, as demonstrated by Yim *et al.* (CSD TAKWIM; Yim *et al.*, 2003 ▸). The coordination pattern of di­thio­ethers is also present in copper halide coordination polymers. These types of organometallic polymers are of significant inter­est due to their intense luminescent properties (Harvey & Knorr, 2010 ▸). In particular, copper(I) halides coordinated to bis­(aryl­thio)­methane ligands (Knorr *et al.*, 2015 ▸). For bromide-bridged copper(I) complexes featuring a *S,N*-donor set, apart from two Cu^II^ species (Al-Noaimi *et al.*, 2024 ▸), just a single match was found, namely bis­(μ-bromo)­bis­{1,3-dimethyl-*N*-[2-(phenyl­sulfan­yl)phen­yl]imidazolidin-2-imine}­dicopper with *d*(Cu⋯Cu) = 2.7545 (5) Å (CSD HUWWIH0; Flörke *et al.*, 2016 ▸). For examples of rhomboid-shaped discrete Cu(μ_2_-I)Cu complexes featuring mixed *N,O* or *P,O* donors sets, see CSD refcodes POHKED (Rofouei & Hashempur, 2008 ▸), BEMBAZ (Shou *et al.*, 2017 ▸) and XOVROR (Kinghat *et al.* 2014 ▸).

## 5. Synthesis and crystallization

Di-μ-iodido-bis­[(1-{[dimeth­yl(phenyl­thio­meth­yl)sil­yl]methyl}iperidine-*k*^2^N^1^,S^1^)copper(I)] (**2a**) was synthesized by adding ligand **1** (614 mg, 2.2 mmol, 1.10 eq.) to CuI (380 mg, 2 mmol, 1.00 eq.) dissolved in aceto­nitrile (8 mL) at room temperature. For complete crystallization the sample was stored for one day at 278 K. Compound **2a** was isolated in the form of colorless blocks. Accordingly, di-μ-bromido-bis­[(1-{[dimeth­yl(phenyl­thio­meth­yl)sil­yl]meth­yl}piperidine-*k*^2^N^1^,S^1^)copper(I)] (**2b**) was prepared by adding **1** (614 mg, 2.2 mmol, 1.10 eq.) to CuBr (286 mg, 2.0 mmol, 1.00 eq.) dissolved in 8 mL aceto­nitrile at room temperature. The sample was also stored at 278 K for one day, allowing the isolation of **2b** as colorless blocks.

## 6. Refinement

Crystal data, data collection and structure refinement details are summarized in Table 3 ▸. H atoms were positioned geometrically (C—H = 0.95–1.00 Å) and refined using a riding model, with *U*_iso_(H) = 1.2*U*_eq_(C) for CH_2_ and CH hydrogen atoms and *U*_iso_(H) = 1.5*U*_eq_(C) for CH_3_ hydrogen atoms. Idealized methyl groups were refined as rotating groups.

## Supplementary Material

Crystal structure: contains datablock(s) 2a, 2b, New_Global_Publ_Block. DOI: 10.1107/S2056989026009862/zn2050sup1.cif

Structure factors: contains datablock(s) 2a. DOI: 10.1107/S2056989026009862/zn20502asup2.hkl

Structure factors: contains datablock(s) 2b. DOI: 10.1107/S2056989026009862/zn20502bsup3.hkl

CCDC references: 2590083, 2590084

Additional supporting information:  crystallographic information; 3D view; checkCIF report

## Figures and Tables

**Table 1:** Selected geometric parameters (Å, °) for **2a**[Chem scheme1]

I1—Cu1	2.5940 (3)	Cu1—S1	2.3533 (6)
I1—Cu1^i^	2.6445 (3)	S1—C7	1.792 (2)
Cu1⋯Cu1^i^	2.8249 (5)		
			
N1—Cu1—S1	97.68 (5)	C10—N1—Cu1	107.94 (12)
N1—Cu1—I1	122.78 (5)	C10—N1—C15	109.23 (15)
N1—Cu1—I1^i^	103.18 (5)	C11—N1—Cu1	110.19 (12)
S1—Cu1—I1	108.475 (16)	C11—N1—C10	107.69 (16)
S1—Cu1—I1^i^	108.077 (16)	C11—N1—C15	108.24 (16)
I1—Cu1—I1^i^	114.743 (10)	C15—N1—Cu1	113.40 (13)
			
Cu1^i^—I1—Cu1—I1^i^	0	Cu1^i^—I1^i^—Cu1—I1	0

Symmetry code: (i) 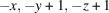.

**Table 2:** Selected geometric parameters (Å, °) for **2b**[Chem scheme1]

Br1—Cu1	2.5197 (4)	Cu1—S1	2.3208 (6)
Br1—Cu1^i^	2.4416 (4)	S1—C7	1.798 (2)
Cu1⋯Cu1^i^	2.7631 (6)		
			
N1—Cu1—S1	98.95 (5)	C10—N1—Cu1	108.05 (13)
N1—Cu1—Br1	100.62 (5)	C11—N1—Cu1	110.20 (13)
N1—Cu1—Br1^i^	123.97 (5)	C11—N1—C10	107.77 (16)
S1—Cu1—Br1	109.379 (19)	C15—N1—Cu1	112.46 (13)
S1—Cu1—Br1^i^	110.223 (18)	C15—N1—C10	109.70 (17)
Br1^i^—Cu1—Br1	112.332 (14)	C15—N1—C11	108.56 (17)
			
Cu1^ii^—Br1—Cu1—I1^ii^	0	Cu1^ii^—Br1^ii^—Cu1—I1	0

Symmetry codes: (i) 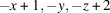; (ii) 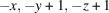.

**Table 3:** Experimental details

	**2a**	**2b**
Crystal data		
Chemical formula	[Cu_2_I_2_(C_15_H_25_NSSi)_2_]	[Cu_2_Br_2_(C_15_H_25_NSSi)_2_]
*M*_r_	939.90	845.92
Crystal system, space group	Triclinic, *P* 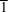	Triclinic, *P* 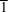
Temperature (K)	173	173
*a*, *b*, *c* (Å)	9.9789 (4), 10.0149 (4), 10.1038 (5)	9.8697 (9), 9.9427 (6), 10.0331 (6)
α, β, γ (°)	74.788 (4), 74.367 (4), 73.590 (4)	73.450 (5), 73.529 (6), 73.946 (6)
*V* (Å^3^)	913.64 (7)	884.28 (12)
*Z*	1	1
Radiation type	Mo *K*α	Mo *K*α
μ (mm^−1^)	3.06	3.67
Crystal size (mm)	0.2 × 0.1 × 0.1	0.2 × 0.1 × 0.1

Data collection		
Diffractometer	Xcalibur, Sapphire3	Xcalibur, Sapphire3
Absorption correction	Multi-scan (*CrysAlis PRO*, Agilent, 2014 ▸)	Multi-scan (*CrysAlis PRO*, Agilent, 2014 ▸)
*T*_min_, *T*_max_	0.866, 1.000	0.815, 1.000
No. of measured, independent and observed [*I* > 2σ(*I*)] reflections	13850, 4322, 4000	11578, 3479, 3121
*R*_int_	0.024	0.034
(sin θ/λ)_max_ (Å^−1^)	0.686	0.617

Refinement		
*R*[*F*^2^ > 2σ(*F*^2^)], *wR*(*F*^2^), *S*	0.021, 0.046, 1.07	0.024, 0.056, 1.05
No. of reflections	4322	3479
No. of parameters	183	183
H-atom treatment	H-atom parameters constrained	H-atom parameters constrained
Δρ_max_, Δρ_min_ (e Å^−3^)	0.52, −0.37	0.39, −0.32

Computer programs: *CrysAlis PRO* (Agilent, 2014 ▸), *SHELXT* (Sheldrick, 2015*a* ▸), *SHELXL* (Sheldrick, 2015*b* ▸), *ORTEP-3 for Windows* (Farrugia, 2012 ▸), *OLEX2* (Dolomanov *et al.*, 2009 ▸), *CrystalExplorer21* (Spackman *et al.* 2021 ▸) and *publCIF* (Westrip, 2010 ▸).

## supplementary crystallographic information

### Di-µ-bromido-bis[(1-{[dimethyl(phenylthiomethyl)silyl]methyl}piperidine-κ2N,S)copper(I)] (2a) Crystal data

**Table:**

[Cu_2_I_2_(C_15_H_25_NSSi)_2_]	*Z* = 1
*M**_r_* = 939.90	*F*(000) = 468
Triclinic, *P*1	*D*_x_ = 1.708 Mg m^−^^3^
*a* = 9.9789 (4) Å	Mo *K*α radiation, λ = 0.71073 Å
*b* = 10.0149 (4) Å	Cell parameters from 4665 reflections
*c* = 10.1038 (5) Å	θ = 2.7–29.0°
α = 74.788 (4)°	µ = 3.06 mm^−^^1^
β = 74.367 (4)°	*T* = 173 K
γ = 73.590 (4)°	Block, colourless
*V* = 913.64 (7) Å^3^	0.2 × 0.1 × 0.1 mm

### Di-µ-bromido-bis[(1-{[dimethyl(phenylthiomethyl)silyl]methyl}piperidine-κ2N,S)copper(I)] (2a) Data collection

**Table:**

Xcalibur, Sapphire3 diffractometer	4322 independent reflections
Radiation source: Enhance (Mo) X-ray Source	4000 reflections with *I* > 2σ(*I*)
Graphite monochromator	*R*_int_ = 0.024
Detector resolution: 16.0560 pixels mm^-1^	θ_max_ = 29.2°, θ_min_ = 2.2°
ω scans	*h* = −13→13
Absorption correction: multi-scan (CrysAlisPro, Agilent, 2014)	*k* = −12→12
*T*_min_ = 0.866, *T*_max_ = 1.000	*l* = −13→13
13850 measured reflections	

### Di-µ-bromido-bis[(1-{[dimethyl(phenylthiomethyl)silyl]methyl}piperidine-κ2N,S)copper(I)] (2a) Refinement

**Table:**

Refinement on *F*^2^	Primary atom site location: dual
Least-squares matrix: full	Hydrogen site location: inferred from neighbouring sites
*R*[*F*^2^ > 2σ(*F*^2^)] = 0.021	H-atom parameters constrained
*wR*(*F*^2^) = 0.046	*w* = 1/[σ^2^(*F*_o_^2^) + (0.0167*P*)^2^ + 0.3459*P*] where *P* = (*F*_o_^2^ + 2*F*_c_^2^)/3
*S* = 1.07	(Δ/σ)_max_ = 0.001
4322 reflections	Δρ_max_ = 0.52 e Å^−^^3^
183 parameters	Δρ_min_ = −0.37 e Å^−^^3^
0 restraints	

### Di-µ-bromido-bis[(1-{[dimethyl(phenylthiomethyl)silyl]methyl}piperidine-κ2N,S)copper(I)] (2a) Special details

**Table:**

Geometry. All esds (except the esd in the dihedral angle between two l.s. planes) are estimated using the full covariance matrix. The cell esds are taken into account individually in the estimation of esds in distances, angles and torsion angles; correlations between esds in cell parameters are only used when they are defined by crystal symmetry. An approximate (isotropic) treatment of cell esds is used for estimating esds involving l.s. planes.

### Di-µ-bromido-bis[(1-{[dimethyl(phenylthiomethyl)silyl]methyl}piperidine-κ2N,S)copper(I)] (2a) Fractional atomic coordinates and isotropic or equivalent isotropic displacement parameters (Å^2^)

**Table:**

	*x*	*y*	*z*	*U*_iso_*/*U*_eq_	
I1	0.06780 (2)	0.31973 (2)	0.66613 (2)	0.01743 (5)	
Cu1	0.14416 (3)	0.50933 (3)	0.44902 (3)	0.01735 (6)	
S1	0.25677 (5)	0.64989 (5)	0.51718 (5)	0.01584 (11)	
Si1	0.37208 (6)	0.74081 (6)	0.20604 (6)	0.01785 (13)	
N1	0.30834 (17)	0.45600 (18)	0.27033 (18)	0.0158 (4)	
C1	0.1595 (2)	0.7237 (2)	0.6666 (2)	0.0159 (4)	
C2	0.1194 (2)	0.8681 (2)	0.6652 (2)	0.0215 (5)	
H2	0.138647	0.934342	0.579854	0.026*	
C3	0.0510 (2)	0.9157 (2)	0.7887 (2)	0.0259 (5)	
H3	0.024847	1.014691	0.787903	0.031*	
C4	0.0207 (2)	0.8207 (2)	0.9126 (2)	0.0243 (5)	
H4	−0.026991	0.854107	0.996773	0.029*	
C5	0.0600 (2)	0.6769 (2)	0.9138 (2)	0.0220 (5)	
H5	0.039332	0.611119	0.999112	0.026*	
C6	0.1292 (2)	0.6278 (2)	0.7914 (2)	0.0191 (4)	
H6	0.155968	0.528652	0.792739	0.023*	
C7	0.2573 (2)	0.7982 (2)	0.3721 (2)	0.0178 (4)	
H7A	0.294285	0.870873	0.392614	0.021*	
H7B	0.158342	0.841553	0.358996	0.021*	
C8	0.3348 (3)	0.8914 (3)	0.0575 (2)	0.0280 (5)	
H8A	0.349905	0.977373	0.074527	0.042*	
H8B	0.235479	0.907385	0.049422	0.042*	
H8C	0.399209	0.868585	−0.029822	0.042*	
C9	0.5620 (2)	0.7058 (3)	0.2169 (3)	0.0288 (5)	
H9A	0.623247	0.661353	0.139147	0.043*	
H9B	0.576520	0.641839	0.306276	0.043*	
H9C	0.586400	0.795547	0.211184	0.043*	
C10	0.3219 (2)	0.5896 (2)	0.1665 (2)	0.0179 (4)	
H10A	0.229140	0.629380	0.137285	0.022*	
H10B	0.393116	0.562221	0.082991	0.022*	
C11	0.2662 (2)	0.3637 (2)	0.2033 (2)	0.0222 (5)	
H11A	0.336944	0.349938	0.115239	0.027*	
H11B	0.172338	0.411965	0.178583	0.027*	
C12	0.2562 (2)	0.2195 (2)	0.2968 (3)	0.0256 (5)	
H12A	0.232010	0.160950	0.245354	0.031*	
H12B	0.178821	0.232026	0.380775	0.031*	
C13	0.3966 (2)	0.1432 (2)	0.3423 (3)	0.0278 (5)	
H13A	0.383521	0.056052	0.413550	0.033*	
H13B	0.470069	0.114689	0.260604	0.033*	
C14	0.4464 (2)	0.2396 (2)	0.4035 (2)	0.0221 (5)	
H14A	0.381405	0.253883	0.494406	0.026*	
H14B	0.543355	0.193165	0.421308	0.026*	
C15	0.4494 (2)	0.3824 (2)	0.3059 (2)	0.0199 (5)	
H15A	0.479042	0.442933	0.350873	0.024*	
H15B	0.521309	0.368636	0.218482	0.024*	

### Di-µ-bromido-bis[(1-{[dimethyl(phenylthiomethyl)silyl]methyl}piperidine-κ2N,S)copper(I)] (2a) Atomic displacement parameters (Å^2^)

**Table:**

	*U*^11^	*U*^22^	*U*^33^	*U*^12^	*U*^13^	*U*^23^
I1	0.01694 (7)	0.01668 (8)	0.01766 (8)	−0.00597 (5)	−0.00423 (5)	0.00114 (5)
Cu1	0.01938 (13)	0.01682 (13)	0.01575 (13)	−0.00612 (10)	−0.00242 (10)	−0.00227 (10)
S1	0.0175 (2)	0.0163 (3)	0.0149 (3)	−0.0056 (2)	−0.00332 (19)	−0.0033 (2)
Si1	0.0171 (3)	0.0197 (3)	0.0156 (3)	−0.0066 (2)	−0.0014 (2)	−0.0012 (2)
N1	0.0154 (8)	0.0190 (9)	0.0150 (9)	−0.0044 (7)	−0.0045 (7)	−0.0051 (7)
C1	0.0158 (10)	0.0211 (11)	0.0136 (10)	−0.0055 (8)	−0.0053 (8)	−0.0047 (8)
C2	0.0293 (12)	0.0163 (11)	0.0182 (11)	−0.0075 (9)	−0.0055 (9)	0.0007 (9)
C3	0.0352 (13)	0.0166 (11)	0.0256 (12)	−0.0024 (10)	−0.0065 (10)	−0.0073 (10)
C4	0.0305 (12)	0.0256 (12)	0.0179 (11)	−0.0049 (10)	−0.0052 (9)	−0.0079 (10)
C5	0.0293 (12)	0.0219 (12)	0.0168 (11)	−0.0097 (9)	−0.0062 (9)	−0.0013 (9)
C6	0.0242 (11)	0.0156 (10)	0.0191 (11)	−0.0057 (9)	−0.0079 (9)	−0.0012 (9)
C7	0.0217 (11)	0.0172 (10)	0.0154 (10)	−0.0091 (9)	−0.0034 (8)	−0.0003 (8)
C8	0.0324 (13)	0.0267 (13)	0.0203 (12)	−0.0077 (10)	−0.0002 (10)	−0.0013 (10)
C9	0.0217 (12)	0.0346 (14)	0.0306 (14)	−0.0100 (10)	−0.0038 (10)	−0.0050 (11)
C10	0.0160 (10)	0.0236 (11)	0.0136 (10)	−0.0041 (9)	−0.0026 (8)	−0.0036 (9)
C11	0.0246 (11)	0.0275 (12)	0.0187 (11)	−0.0076 (9)	−0.0044 (9)	−0.0100 (10)
C12	0.0310 (12)	0.0249 (12)	0.0273 (13)	−0.0110 (10)	−0.0061 (10)	−0.0111 (10)
C13	0.0323 (13)	0.0197 (12)	0.0328 (14)	−0.0062 (10)	−0.0064 (10)	−0.0078 (10)
C14	0.0191 (11)	0.0195 (11)	0.0267 (12)	−0.0018 (9)	−0.0075 (9)	−0.0033 (10)
C15	0.0151 (10)	0.0208 (11)	0.0249 (12)	−0.0038 (9)	−0.0055 (8)	−0.0052 (9)

### Di-µ-bromido-bis[(1-{[dimethyl(phenylthiomethyl)silyl]methyl}piperidine-κ2N,S)copper(I)] (2a) Geometric parameters (Å, º)

**Table:**

I1—Cu1	2.5940 (3)	C7—H7A	0.9900
I1—Cu1^i^	2.6445 (3)	C7—H7B	0.9900
Cu1—Cu1^i^	2.8249 (5)	C8—H8A	0.9800
Cu1—S1	2.3533 (6)	C8—H8B	0.9800
Cu1—N1	2.1556 (17)	C8—H8C	0.9800
S1—C1	1.777 (2)	C9—H9A	0.9800
S1—C7	1.792 (2)	C9—H9B	0.9800
Si1—C7	1.886 (2)	C9—H9C	0.9800
Si1—C8	1.861 (2)	C10—H10A	0.9900
Si1—C9	1.855 (2)	C10—H10B	0.9900
Si1—C10	1.886 (2)	C11—H11A	0.9900
N1—C10	1.483 (3)	C11—H11B	0.9900
N1—C11	1.480 (3)	C11—C12	1.517 (3)
N1—C15	1.484 (3)	C12—H12A	0.9900
C1—C2	1.385 (3)	C12—H12B	0.9900
C1—C6	1.388 (3)	C12—C13	1.522 (3)
C2—H2	0.9500	C13—H13A	0.9900
C2—C3	1.385 (3)	C13—H13B	0.9900
C3—H3	0.9500	C13—C14	1.521 (3)
C3—C4	1.377 (3)	C14—H14A	0.9900
C4—H4	0.9500	C14—H14B	0.9900
C4—C5	1.380 (3)	C14—C15	1.512 (3)
C5—H5	0.9500	C15—H15A	0.9900
C5—C6	1.384 (3)	C15—H15B	0.9900
C6—H6	0.9500		
			
N1—Cu1—S1	97.68 (5)	H7A—C7—H7B	108.1
N1—Cu1—I1	122.78 (5)	Si1—C8—H8A	109.5
N1—Cu1—I1^i^	103.18 (5)	Si1—C8—H8B	109.5
S1—Cu1—I1	108.475 (16)	Si1—C8—H8C	109.5
S1—Cu1—I1^i^	108.077 (16)	H8A—C8—H8B	109.5
Cu1—I1—Cu1^i^	65.257 (10)	H8A—C8—H8C	109.5
I1—Cu1—I1^i^	114.743 (10)	H8B—C8—H8C	109.5
I1^i^—Cu1—Cu1^i^	56.508 (10)	Si1—C9—H9A	109.5
I1—Cu1—Cu1^i^	58.234 (10)	Si1—C9—H9B	109.5
S1—Cu1—Cu1^i^	125.555 (19)	Si1—C9—H9C	109.5
N1—Cu1—Cu1^i^	135.29 (5)	H9A—C9—H9B	109.5
C1—S1—Cu1	115.60 (7)	H9A—C9—H9C	109.5
C1—S1—C7	105.22 (10)	H9B—C9—H9C	109.5
C7—S1—Cu1	101.58 (7)	Si1—C10—H10A	106.6
C8—Si1—C7	107.28 (10)	Si1—C10—H10B	106.6
C8—Si1—C10	104.34 (10)	N1—C10—Si1	122.89 (14)
C9—Si1—C7	107.99 (10)	N1—C10—H10A	106.6
C9—Si1—C8	109.50 (11)	N1—C10—H10B	106.6
C9—Si1—C10	114.44 (11)	H10A—C10—H10B	106.6
C10—Si1—C7	113.00 (9)	N1—C11—H11A	109.0
C10—N1—Cu1	107.94 (12)	N1—C11—H11B	109.0
C10—N1—C15	109.23 (15)	N1—C11—C12	112.88 (18)
C11—N1—Cu1	110.19 (12)	H11A—C11—H11B	107.8
C11—N1—C10	107.69 (16)	C12—C11—H11A	109.0
C11—N1—C15	108.24 (16)	C12—C11—H11B	109.0
C15—N1—Cu1	113.40 (13)	C11—C12—H12A	109.4
C2—C1—S1	123.74 (17)	C11—C12—H12B	109.4
C2—C1—C6	119.77 (19)	C11—C12—C13	111.07 (18)
C6—C1—S1	116.43 (16)	H12A—C12—H12B	108.0
C1—C2—H2	120.1	C13—C12—H12A	109.4
C1—C2—C3	119.7 (2)	C13—C12—H12B	109.4
C3—C2—H2	120.1	C12—C13—H13A	109.6
C2—C3—H3	119.7	C12—C13—H13B	109.6
C4—C3—C2	120.6 (2)	H13A—C13—H13B	108.1
C4—C3—H3	119.7	C14—C13—C12	110.35 (19)
C3—C4—H4	120.2	C14—C13—H13A	109.6
C3—C4—C5	119.6 (2)	C14—C13—H13B	109.6
C5—C4—H4	120.2	C13—C14—H14A	109.3
C4—C5—H5	119.8	C13—C14—H14B	109.3
C4—C5—C6	120.4 (2)	H14A—C14—H14B	107.9
C6—C5—H5	119.8	C15—C14—C13	111.69 (19)
C1—C6—H6	120.1	C15—C14—H14A	109.3
C5—C6—C1	119.9 (2)	C15—C14—H14B	109.3
C5—C6—H6	120.1	N1—C15—C14	112.25 (17)
S1—C7—Si1	110.86 (11)	N1—C15—H15A	109.2
S1—C7—H7A	109.5	N1—C15—H15B	109.2
S1—C7—H7B	109.5	C14—C15—H15A	109.2
Si1—C7—H7A	109.5	C14—C15—H15B	109.2
Si1—C7—H7B	109.5	H15A—C15—H15B	107.9
			
Cu1—S1—C1—C2	−121.35 (17)	C7—Si1—C10—N1	−51.87 (19)
Cu1—S1—C1—C6	61.48 (17)	C8—Si1—C7—S1	168.04 (11)
Cu1—S1—C7—Si1	−64.61 (10)	C8—Si1—C10—N1	−168.07 (16)
Cu1—N1—C10—Si1	60.38 (17)	C9—Si1—C7—S1	−74.02 (14)
Cu1—N1—C11—C12	−65.5 (2)	C9—Si1—C10—N1	72.28 (19)
Cu1—N1—C15—C14	63.7 (2)	C10—Si1—C7—S1	53.60 (14)
S1—C1—C2—C3	−176.20 (17)	C10—N1—C11—C12	176.99 (17)
S1—C1—C6—C5	176.87 (16)	C10—N1—C15—C14	−175.84 (17)
N1—C11—C12—C13	−56.6 (2)	C11—N1—C10—Si1	179.33 (14)
C1—S1—C7—Si1	174.54 (10)	C11—N1—C15—C14	−58.8 (2)
C1—C2—C3—C4	−1.0 (3)	C11—C12—C13—C14	51.3 (3)
C2—C1—C6—C5	−0.4 (3)	C12—C13—C14—C15	−51.8 (2)
C2—C3—C4—C5	0.6 (4)	C13—C14—C15—N1	56.9 (2)
C3—C4—C5—C6	−0.1 (3)	C15—N1—C10—Si1	−63.3 (2)
C4—C5—C6—C1	0.0 (3)	C15—N1—C11—C12	59.0 (2)
C6—C1—C2—C3	0.9 (3)	Cu1^i^—I1—Cu1—I1^i^	0
C7—S1—C1—C2	−10.2 (2)	Cu1^i^—I1^i^—Cu1—I1	0
C7—S1—C1—C6	172.64 (16)		

Symmetry code: (i) −*x*, −*y*+1, −*z*+1.

### Di-µ-iodido-bis[(1-{[dimethyl(phenylthiomethyl)silyl]methyl}piperidine-κ2N,S)copper(I)] (2b) Crystal data

**Table:**

[Cu_2_Br_2_(C_15_H_25_NSSi)_2_]	*Z* = 1
*M**_r_* = 845.92	*F*(000) = 432
Triclinic, *P*1	*D*_x_ = 1.588 Mg m^−^^3^
*a* = 9.8697 (9) Å	Mo *K*α radiation, λ = 0.71073 Å
*b* = 9.9427 (6) Å	Cell parameters from 5514 reflections
*c* = 10.0331 (6) Å	θ = 2.7–29.0°
α = 73.450 (5)°	µ = 3.67 mm^−^^1^
β = 73.529 (6)°	*T* = 173 K
γ = 73.946 (6)°	Block, colourless
*V* = 884.28 (12) Å^3^	0.2 × 0.1 × 0.1 mm

### Di-µ-iodido-bis[(1-{[dimethyl(phenylthiomethyl)silyl]methyl}piperidine-κ2N,S)copper(I)] (2b) Data collection

**Table:**

Xcalibur, Sapphire3 diffractometer	3479 independent reflections
Radiation source: Enhance (Mo) X-ray Source	3121 reflections with *I* > 2σ(*I*)
Graphite monochromator	*R*_int_ = 0.034
Detector resolution: 16.0560 pixels mm^-1^	θ_max_ = 26.0°, θ_min_ = 2.2°
ω scans	*h* = −12→12
Absorption correction: multi-scan (CrysAlisPro, Agilent, 2014)	*k* = −12→12
*T*_min_ = 0.815, *T*_max_ = 1.000	*l* = −12→12
11578 measured reflections	

### Di-µ-iodido-bis[(1-{[dimethyl(phenylthiomethyl)silyl]methyl}piperidine-κ2N,S)copper(I)] (2b) Refinement

**Table:**

Refinement on *F*^2^	Primary atom site location: iterative
Least-squares matrix: full	Hydrogen site location: inferred from neighbouring sites
*R*[*F*^2^ > 2σ(*F*^2^)] = 0.024	H-atom parameters constrained
*wR*(*F*^2^) = 0.056	*w* = 1/[σ^2^(*F*_o_^2^) + (0.0217*P*)^2^ + 0.1872*P*] where *P* = (*F*_o_^2^ + 2*F*_c_^2^)/3
*S* = 1.05	(Δ/σ)_max_ = 0.001
3479 reflections	Δρ_max_ = 0.39 e Å^−^^3^
183 parameters	Δρ_min_ = −0.32 e Å^−^^3^
0 restraints	

### Di-µ-iodido-bis[(1-{[dimethyl(phenylthiomethyl)silyl]methyl}piperidine-κ2N,S)copper(I)] (2b) Special details

**Table:**

Geometry. All esds (except the esd in the dihedral angle between two l.s. planes) are estimated using the full covariance matrix. The cell esds are taken into account individually in the estimation of esds in distances, angles and torsion angles; correlations between esds in cell parameters are only used when they are defined by crystal symmetry. An approximate (isotropic) treatment of cell esds is used for estimating esds involving l.s. planes.

### Di-µ-iodido-bis[(1-{[dimethyl(phenylthiomethyl)silyl]methyl}piperidine-κ2N,S)copper(I)] (2b) Fractional atomic coordinates and isotropic or equivalent isotropic displacement parameters (Å^2^)

**Table:**

	*x*	*y*	*z*	*U*_iso_*/*U*_eq_	
Br1	0.44330 (2)	0.16667 (2)	0.83511 (2)	0.01766 (7)	
Cu1	0.64346 (3)	0.01004 (3)	0.95483 (3)	0.01744 (8)	
S1	0.75500 (6)	0.15336 (6)	1.01668 (6)	0.01535 (13)	
Si1	0.87510 (7)	0.24369 (7)	0.69886 (6)	0.01714 (15)	
N1	0.80590 (19)	−0.04401 (19)	0.77416 (18)	0.0144 (4)	
C1	0.6580 (2)	0.2289 (2)	1.1663 (2)	0.0149 (5)	
C2	0.6241 (3)	0.3746 (2)	1.1623 (2)	0.0203 (5)	
H2	0.646215	0.441359	1.074519	0.024*	
C3	0.5580 (3)	0.4226 (3)	1.2866 (2)	0.0237 (6)	
H3	0.536757	0.522468	1.283898	0.028*	
C4	0.5225 (3)	0.3271 (3)	1.4146 (2)	0.0234 (6)	
H4	0.476526	0.360868	1.499371	0.028*	
C5	0.5546 (3)	0.1816 (3)	1.4181 (2)	0.0200 (5)	
H5	0.530215	0.115342	1.505759	0.024*	
C6	0.6222 (2)	0.1319 (2)	1.2946 (2)	0.0181 (5)	
H6	0.643874	0.032017	1.297541	0.022*	
C7	0.7568 (3)	0.3027 (2)	0.8650 (2)	0.0186 (5)	
H7A	0.793498	0.377756	0.882548	0.022*	
H7B	0.657043	0.344529	0.851143	0.022*	
C8	1.0676 (3)	0.2078 (3)	0.7091 (3)	0.0259 (6)	
H8A	1.129729	0.165515	0.629573	0.039*	
H8B	1.081440	0.140825	0.799549	0.039*	
H8C	1.093103	0.298004	0.703684	0.039*	
C9	0.8400 (3)	0.3960 (3)	0.5453 (2)	0.0266 (6)	
H9A	0.852695	0.483819	0.561937	0.040*	
H9B	0.740823	0.409670	0.535477	0.040*	
H9C	0.908085	0.374756	0.457758	0.040*	
C10	0.8212 (2)	0.0911 (2)	0.6644 (2)	0.0167 (5)	
H10A	0.727342	0.131098	0.635901	0.020*	
H10B	0.892652	0.063205	0.579862	0.020*	
C11	0.7603 (3)	−0.1381 (2)	0.7116 (2)	0.0199 (5)	
H11A	0.832123	−0.154694	0.623141	0.024*	
H11B	0.666236	−0.088987	0.685868	0.024*	
C12	0.7457 (3)	−0.2822 (2)	0.8139 (3)	0.0225 (5)	
H12A	0.667195	−0.266790	0.898499	0.027*	
H12B	0.719428	−0.342937	0.766290	0.027*	
C13	0.8855 (3)	−0.3588 (3)	0.8609 (3)	0.0246 (6)	
H13A	0.959846	−0.389626	0.779328	0.030*	
H13B	0.869890	−0.445559	0.936751	0.030*	
C14	0.9384 (3)	−0.2601 (2)	0.9167 (2)	0.0207 (5)	
H14A	0.871620	−0.242401	1.007412	0.025*	
H14B	1.035159	−0.307698	0.936562	0.025*	
C15	0.9471 (2)	−0.1180 (2)	0.8106 (2)	0.0179 (5)	
H15A	0.979252	−0.055672	0.851440	0.021*	
H15B	1.019779	−0.135032	0.722678	0.021*	

### Di-µ-iodido-bis[(1-{[dimethyl(phenylthiomethyl)silyl]methyl}piperidine-κ2N,S)copper(I)] (2b) Atomic displacement parameters (Å^2^)

**Table:**

	*U*^11^	*U*^22^	*U*^33^	*U*^12^	*U*^13^	*U*^23^
Br1	0.01788 (13)	0.01598 (12)	0.01710 (13)	−0.00516 (10)	−0.00396 (10)	0.00099 (9)
Cu1	0.01935 (16)	0.01641 (15)	0.01617 (15)	−0.00650 (12)	−0.00149 (13)	−0.00287 (12)
S1	0.0178 (3)	0.0148 (3)	0.0142 (3)	−0.0049 (2)	−0.0034 (2)	−0.0031 (2)
Si1	0.0182 (3)	0.0174 (3)	0.0147 (3)	−0.0063 (3)	−0.0020 (3)	−0.0010 (3)
N1	0.0137 (10)	0.0153 (9)	0.0152 (9)	−0.0038 (8)	−0.0042 (8)	−0.0037 (8)
C1	0.0134 (11)	0.0194 (12)	0.0146 (11)	−0.0040 (10)	−0.0058 (10)	−0.0051 (9)
C2	0.0246 (13)	0.0180 (12)	0.0178 (12)	−0.0046 (10)	−0.0059 (11)	−0.0021 (10)
C3	0.0300 (14)	0.0147 (12)	0.0276 (13)	−0.0026 (11)	−0.0077 (12)	−0.0072 (10)
C4	0.0259 (14)	0.0270 (14)	0.0189 (12)	−0.0011 (11)	−0.0061 (11)	−0.0106 (11)
C5	0.0240 (13)	0.0222 (13)	0.0143 (11)	−0.0062 (11)	−0.0062 (10)	−0.0014 (10)
C6	0.0222 (13)	0.0154 (12)	0.0192 (12)	−0.0068 (10)	−0.0071 (10)	−0.0026 (9)
C7	0.0225 (13)	0.0171 (12)	0.0170 (12)	−0.0083 (10)	−0.0040 (10)	−0.0014 (9)
C8	0.0226 (13)	0.0297 (14)	0.0256 (13)	−0.0115 (11)	−0.0026 (11)	−0.0037 (11)
C9	0.0310 (15)	0.0231 (13)	0.0209 (13)	−0.0092 (12)	−0.0022 (12)	0.0023 (11)
C10	0.0175 (12)	0.0197 (12)	0.0122 (11)	−0.0047 (10)	−0.0019 (10)	−0.0030 (9)
C11	0.0228 (13)	0.0232 (13)	0.0190 (12)	−0.0059 (11)	−0.0073 (11)	−0.0092 (10)
C12	0.0279 (14)	0.0202 (13)	0.0255 (13)	−0.0084 (11)	−0.0070 (11)	−0.0101 (10)
C13	0.0285 (14)	0.0174 (12)	0.0291 (14)	−0.0067 (11)	−0.0058 (12)	−0.0057 (11)
C14	0.0172 (12)	0.0194 (12)	0.0253 (13)	−0.0031 (10)	−0.0080 (11)	−0.0019 (10)
C15	0.0138 (12)	0.0178 (12)	0.0228 (12)	−0.0028 (10)	−0.0051 (10)	−0.0052 (10)

### Di-µ-iodido-bis[(1-{[dimethyl(phenylthiomethyl)silyl]methyl}piperidine-κ2N,S)copper(I)] (2b) Geometric parameters (Å, º)

**Table:**

Br1—Cu1	2.5197 (4)	C7—H7A	0.9900
Br1—Cu1^i^	2.4416 (4)	C7—H7B	0.9900
Cu1—Cu1^i^	2.7631 (6)	C8—H8A	0.9800
Cu1—S1	2.3208 (6)	C8—H8B	0.9800
Cu1—N1	2.1391 (18)	C8—H8C	0.9800
S1—C1	1.778 (2)	C9—H9A	0.9800
S1—C7	1.798 (2)	C9—H9B	0.9800
Si1—C7	1.887 (2)	C9—H9C	0.9800
Si1—C8	1.861 (2)	C10—H10A	0.9900
Si1—C9	1.863 (2)	C10—H10B	0.9900
Si1—C10	1.885 (2)	C11—H11A	0.9900
N1—C10	1.489 (3)	C11—H11B	0.9900
N1—C11	1.483 (3)	C11—C12	1.523 (3)
N1—C15	1.478 (3)	C12—H12A	0.9900
C1—C2	1.385 (3)	C12—H12B	0.9900
C1—C6	1.391 (3)	C12—C13	1.514 (3)
C2—H2	0.9500	C13—H13A	0.9900
C2—C3	1.384 (3)	C13—H13B	0.9900
C3—H3	0.9500	C13—C14	1.520 (3)
C3—C4	1.381 (3)	C14—H14A	0.9900
C4—H4	0.9500	C14—H14B	0.9900
C4—C5	1.385 (3)	C14—C15	1.515 (3)
C5—H5	0.9500	C15—H15A	0.9900
C5—C6	1.386 (3)	C15—H15B	0.9900
C6—H6	0.9500		
			
N1—Cu1—S1	98.95 (5)	H7A—C7—H7B	108.1
N1—Cu1—Br1	100.62 (5)	Si1—C8—H8A	109.5
N1—Cu1—Br1^i^	123.97 (5)	Si1—C8—H8B	109.5
S1—Cu1—Br1	109.379 (19)	Si1—C8—H8C	109.5
S1—Cu1—Br1^i^	110.223 (18)	H8A—C8—H8B	109.5
Br1^i^—Cu1—Br1	112.332 (14)	H8A—C8—H8C	109.5
Cu1^i^—Br1—Cu1	67.668 (13)	H8B—C8—H8C	109.5
Br1^i^—Cu1—Cu1^i^	57.513 (12)	Si1—C9—H9A	109.5
Br1—Cu1—Cu1^i^	54.819 (12)	Si1—C9—H9B	109.5
S1—Cu1—Cu1^i^	127.45 (2)	Si1—C9—H9C	109.5
N1—Cu1—Cu1^i^	131.44 (5)	H9A—C9—H9B	109.5
C1—S1—Cu1	116.49 (7)	H9A—C9—H9C	109.5
C1—S1—C7	105.35 (10)	H9B—C9—H9C	109.5
C7—S1—Cu1	101.37 (8)	Si1—C10—H10A	106.7
C8—Si1—C7	108.62 (11)	Si1—C10—H10B	106.7
C8—Si1—C9	109.66 (12)	N1—C10—Si1	122.41 (15)
C8—Si1—C10	114.59 (11)	N1—C10—H10A	106.7
C9—Si1—C7	106.74 (11)	N1—C10—H10B	106.7
C9—Si1—C10	104.44 (11)	H10A—C10—H10B	106.6
C10—Si1—C7	112.43 (10)	N1—C11—H11A	109.2
C10—N1—Cu1	108.05 (13)	N1—C11—H11B	109.2
C11—N1—Cu1	110.20 (13)	N1—C11—C12	112.25 (18)
C11—N1—C10	107.77 (16)	H11A—C11—H11B	107.9
C15—N1—Cu1	112.46 (13)	C12—C11—H11A	109.2
C15—N1—C10	109.70 (17)	C12—C11—H11B	109.2
C15—N1—C11	108.56 (17)	C11—C12—H12A	109.4
C2—C1—S1	123.93 (17)	C11—C12—H12B	109.4
C2—C1—C6	119.8 (2)	H12A—C12—H12B	108.0
C6—C1—S1	116.20 (17)	C13—C12—C11	111.02 (19)
C1—C2—H2	120.1	C13—C12—H12A	109.4
C3—C2—C1	119.8 (2)	C13—C12—H12B	109.4
C3—C2—H2	120.1	C12—C13—H13A	109.6
C2—C3—H3	119.6	C12—C13—H13B	109.6
C4—C3—C2	120.7 (2)	C12—C13—C14	110.39 (19)
C4—C3—H3	119.6	H13A—C13—H13B	108.1
C3—C4—H4	120.3	C14—C13—H13A	109.6
C3—C4—C5	119.4 (2)	C14—C13—H13B	109.6
C5—C4—H4	120.3	C13—C14—H14A	109.3
C4—C5—H5	119.8	C13—C14—H14B	109.3
C4—C5—C6	120.4 (2)	H14A—C14—H14B	108.0
C6—C5—H5	119.8	C15—C14—C13	111.55 (19)
C1—C6—H6	120.1	C15—C14—H14A	109.3
C5—C6—C1	119.8 (2)	C15—C14—H14B	109.3
C5—C6—H6	120.1	N1—C15—C14	111.56 (18)
S1—C7—Si1	110.73 (12)	N1—C15—H15A	109.3
S1—C7—H7A	109.5	N1—C15—H15B	109.3
S1—C7—H7B	109.5	C14—C15—H15A	109.3
Si1—C7—H7A	109.5	C14—C15—H15B	109.3
Si1—C7—H7B	109.5	H15A—C15—H15B	108.0
			
Cu1—S1—C1—C2	−124.41 (19)	C7—Si1—C10—N1	−53.0 (2)
Cu1—S1—C1—C6	58.85 (19)	C8—Si1—C7—S1	−72.86 (14)
Cu1—S1—C7—Si1	−64.89 (12)	C8—Si1—C10—N1	71.6 (2)
Cu1—N1—C10—Si1	60.00 (19)	C9—Si1—C7—S1	168.97 (12)
Cu1—N1—C11—C12	−64.1 (2)	C9—Si1—C10—N1	−168.38 (18)
Cu1—N1—C15—C14	62.6 (2)	C10—Si1—C7—S1	55.03 (15)
S1—C1—C2—C3	−175.05 (18)	C10—N1—C11—C12	178.24 (19)
S1—C1—C6—C5	175.94 (17)	C10—N1—C15—C14	−177.15 (17)
N1—C11—C12—C13	−56.6 (3)	C11—N1—C10—Si1	179.07 (16)
C1—S1—C7—Si1	173.31 (11)	C11—N1—C15—C14	−59.6 (2)
C1—C2—C3—C4	−1.3 (4)	C11—C12—C13—C14	51.7 (3)
C2—C1—C6—C5	−0.9 (3)	C12—C13—C14—C15	−52.5 (3)
C2—C3—C4—C5	0.4 (4)	C13—C14—C15—N1	57.5 (3)
C3—C4—C5—C6	0.2 (4)	C15—N1—C10—Si1	−62.9 (2)
C4—C5—C6—C1	0.1 (4)	C15—N1—C11—C12	59.5 (2)
C6—C1—C2—C3	1.6 (4)	Cu1^ii^—Br1—Cu1—I1^ii^	0
C7—S1—C1—C2	−13.0 (2)	Cu1^ii^—Br1^ii^—Cu1—I1	0
C7—S1—C1—C6	170.27 (17)		

Symmetry codes: (i) −*x*+1, −*y*, −*z*+2; (ii) −*x*, −*y*+1, −*z*+1.

### Selected geometric parameters (Å, °) for 2a

**Table:**

I1—Cu1	2.5940 (3)
I1—Cu1i	2.6445 (3)
Cu1···Cu1i	2.8249 (5)
Cu1—S1	2.3533 (6)
S1—C7	1.792 (2)
N1—Cu1—S1	97.68 (5)
N1—Cu1—I1	122.78 (5)
N1—Cu1—I1i	103.18 (5)
S1—Cu1—I1	108.475 (16)
S1—Cu1—I1i	108.077 (16)
I1—Cu1—I1i	114.743 (10)
C10—N1—Cu1	107.94 (12)
C10—N1—C15	109.23 (15)
C11—N1—Cu1	110.19 (12)
C11—N1—C10	107.69 (16)
C11—N1—C15	108.24 (16)
C15—N1—Cu1	113.40 (13)
Cu1i—I1—Cu1—I1i	0.000
Cu1i—I1i—Cu1—I1	0.000

Symmetry code: (i) -*x*, 1 - *y*, 1 - *z*.

### Selected geometric parameters (Å, °) for 2b.

**Table:**

Br1—Cu1	2.5197 (4)
Br1—Cu1i	2.4416 (4)
Cu1···Cu1i	2.7631 (6)
Cu1—S1	2.3208 (6)
S1—C7	1.798 (2)
N1–Cu1—S1	98.95 (5)
N1—Cu1—Br1	100.62 (5)
N1—Cu1—Br1i	123.97 (5)
S1—Cu1—Br1	109.379 (19)
S1—Cu1—Br1i	110.223 (18)
Br1—Cu1—Br1i	112.332 (14)
C10—N1—Cu1	108.05 (13)
C11—N1—Cu1	110.20 (13)
C11—N1—C10	107.77 (16)
C15—N1—Cu1	112.46 (13)
C15—N1—C10	109.70 (17)
C15—N1—C11	108.56 (17)
Cu1i—Br1—Cu1—Br1i	0.000
Cu1i—Br1i—Cu1—Br1	0.000

Symmetry code: (i) 1–x, –y, 2–z
